# Early Neolithic executions indicated by clustered cranial trauma in the mass grave of Halberstadt

**DOI:** 10.1038/s41467-018-04773-w

**Published:** 2018-06-25

**Authors:** Christian Meyer, Corina Knipper, Nicole Nicklisch, Angelina Münster, Olaf Kürbis, Veit Dresely, Harald Meller, Kurt W. Alt

**Affiliations:** 1 State Office for Heritage Management and Archaeology Saxony-Anhalt/State Museum of Prehistory, Richard-Wagner-Str. 9, 06114 Halle (Saale), Germany; 2 OsteoARC, OsteoArchaeological Research Center, Rammelsberger Str. 26, 38644 Goslar, Germany; 3 Institute of Anthropology, University of Mainz, Saarstr. 21, 55099 Mainz, Germany; 4 Curt Engelhorn Centre Archaeometry gGmbH, D6 3, 68159 Mannheim, Germany; 5 Center of Natural and Cultural Human History, Danube Private University, Steiner Landstr. 124, 3500 Krems, Austria; 6 Integrative Prehistory and Archaeological Science, University of Basel, Spalenring 145, 4055 Basel, Switzerland

## Abstract

The later phase of the Central European Early Neolithic witnessed a rise in collective lethal violence to a level undocumented up to this date. This is evidenced by repeated massacres of settled communities of the *Linearbandkeramik* (ca. 5600–4900 cal bc), the first full farming culture in this area. Skeletal remains of several dozen victims of this prehistoric warfare are known from different sites in Germany and Austria. Here we show that the mass grave of Halberstadt, Germany, a new mass fatality site from the same period, reveals further and so far unknown facets of Early Neolithic collective lethal violence. A highly selected, almost exclusively adult male and non-local population sample was killed by targeted blows to the back of the head, indicating a practice of systematic execution under largely controlled conditions followed by careless disposal of the bodies. This discovery significantly increases current knowledge about warfare-related violent behaviour in Early Neolithic Central Europe.

## Abstract (web summary)

Prehistoric warfare and massacres of *Linearbandkeramik* (LBK) communities are evidenced by mass graves from the Early Neolithic of Central Europe. Here, Meyer et al. describe a newly discovered mass grave from Germany revealing the execution of a predominantly adult male group of non-local individuals.

## Introduction

In Central Europe, the first full Neolithic farming culture is the *Linearbandkeramik* (LBK; ca. 5600–4900 cal bc), which widely replaced the Mesolithic foraging way of life. While the LBK has long been considered well-researched^1–3^, recent studies have revealed substantial differences between the genetic compositions of Mesolithic and Early Neolithic populations. These major discontinuities were caused by an influx of Neolithic farmers originating from southeastern Europe, where the LBK itself developed^4–7^. Spreading from there, LBK farmers introduced domesticated animals and crops to Central Europe as the economic basis of a new way of life in this region. With time these new settlers colonised large parts of Central Europe, mainly targeting the fertile loess soils^1, 2^. By firmly establishing the Early Neolithic agricultural system these people significantly changed their natural environment by clearing forests and establishing permanent settlements and farming plots, in which considerable amounts of time and effort were invested^8^. It is assumed that kinship and membership in probably pioneering lineages played an important part in the organisation of LBK communities^2, 3, 9, 10^. Specific patterns known so far from biomolecular studies include differentiation in the funeral rite connected to the place of origin and very likely virilocal residence patterns in addition to the large-scale genetic changes during the Mesolithic–Neolithic transition^4, 9^.

The skeletal remains of the overall LBK population form the biogenic basis of these summary studies, but the specific contexts in which they are discovered also hold key information for characterising their actual life and death experiences as a bioarchaeological record^11^. Detailed analyses of how and why the deceased became part of their respective burial features allow important insights into the complex interplay of actors, factors, and events that shaped the contemporary LBK experience of communities and individuals^12^. Usually, LBK burials are found as individual inhumations or cremations in dedicated cemeteries^13^. Overall, there is a large variety but left-crouched inhumation burials are consistently encountered, often equipped with grave goods. The most common grave orientation is roughly East–West, but extended, right-crouched, unfurnished, or differently orientated burials do occur as well^14^. A substantial part of the LBK skeletal record stems from settlement burials, which do not differ greatly from the cemetery burials^15^. Multiple burials of carefully arranged bodies are rare^16^.

Also found in various LBK contexts are isolated human body parts and partial skeletons. These occur scattered within settlement pits, in natural caves, or in enclosure pits and ditches and might be the remains of disturbed older burials or have had ritualistic significance. This last point is exemplified by the large enclosure site of Herxheim, Germany, where human bodies were subjected to complex postmortem manipulation and disarticulation before being deposited in mixed assemblages with selected animal bones and various artefacts^17^. The highly intense manipulation of corpses and possible ritualistic cannibalism practised at Herxheim represent behaviours that are still difficult to unravel, but knowledge about them has affected the interpretation of other sites that have long been considered as evidence for LBK warfare and, more specifically, massacres^18, 19^. These sites are the mass grave of Talheim, Germany and the enclosure at Asparn/Schletz, Austria^20–23^. Both yielded human skeletal remains bearing clear signs of perimortem lethal violence such as blunt force cranial injuries and possible arrow wounds. While the bodies at Talheim had been quickly collected and deposited in a disorganised mass grave, those at Asparn show signs of carnivore gnawing and partial disarticulation that occurred before natural sedimentation in the ditches^23^. Recently, a third massacre site has been analysed: the mass grave of Schöneck-Kilianstädten, Germany^24^. It is assumed that at these three sites LBK communities were wilfully destroyed by unknown attackers, most likely members of other groups labelled today as LBK, possibly even close neighbours. The recent interpretation of the Kilianstädten mass grave as a further violence-related mass fatality site that shows similar patterns to Talheim and Asparn/Schletz again strengthens the interpretation of these massacres as warfare-related^24^.

Signs of careful funeral ritual, clearly evident in the regular graves of the LBK, are absent for the massacre victims. While some younger women might have been captured rather than killed, which is suggested for all three massacre sites currently known^23–25^, the victimised population samples seem to be representative of settled LBK communities, including balanced numbers of children and adults of both sexes. The high percentage of subadults among the massacre victims and their burial within settlement areas are both strongly indicative of surprise attacks on villages, a practice encountered among many different societies^26, 27^. One further LBK mass grave at Wiederstedt, Germany is even dominated by subadult individuals (80%; *N* = 10), but in the absence of recognisable perimortem injuries another cause of death seems more likely in this case. However, like the other mass graves, it was located in a settlement context^12, 28^, thereby demonstrating again the close association of LBK children with village sites. This is also evident from the settlement burials where they generally dominate^15, 29^.

Lacking from the massacre or mass grave sites is a clearly focussed and patterned postmortem treatment like that found for example at Herxheim. Although targeted destruction of the lower leg bones was observed at Kilianstädten, this apparently occurred within the context of the massacre itself and thereby likely ante-mortem. It does not fit within a framework of complex long-term cyclic actions like at Herxheim. Mutilation and torture are well known to occur as part of massacres, which are clearly defined as being limited in time^30^. Once the victims expired, their physical remains likely held no more meaning for the aggressors and apparently were disposed of without further ritual treatment^12^. This postmortem neglect again suggests that the massacre victims were casualties of intergroup warfare rather than part of complex ritual actions charged with greater meaning^31^.

Into this tableau of LBK collective lethal violence and mass burial we add a new site: the mass grave of Halberstadt, Germany. It contained the skeletal remains of irregularly deposited and severely traumatised individuals. Careful excavation of the block-lifted feature and subsequent laboratory analyses revealed some similarities to the other LBK mass fatality sites, but, more importantly, also significant and currently unique deviations from them. By applying osteological, palaeopathological, taphonomic, and isotope analyses (strontium, carbon and nitrogen), this study adds important and previously unavailable information to the discussion of the scope and frequency of Early Neolithic violence and its contextual interpretation.

## Results

### The archaeological site and feature

The mass grave was discovered in 2013 during archaeological excavations prior to housing development in the southern outskirts of Halberstadt, a town located just north of the Harz mountains in the German state of Saxony-Anhalt (Supplementary Fig. 1). This part of the Halberstadt area, called “Sonntagsfeld”, is rich in archaeological remains from various periods and has been settled since the Early Neolithic; traces of six LBK longhouses have previously been identified here^32^. In the same area, at least 38 regular LBK inhumation burials have been excavated so far, which relate spatially mainly to the houses and therefore are examples of settlement burials. Overall, these graves represent a carefully deployed LBK burial site including subadults and adults of both sexes^32, 33^.

**Fig. 1:**
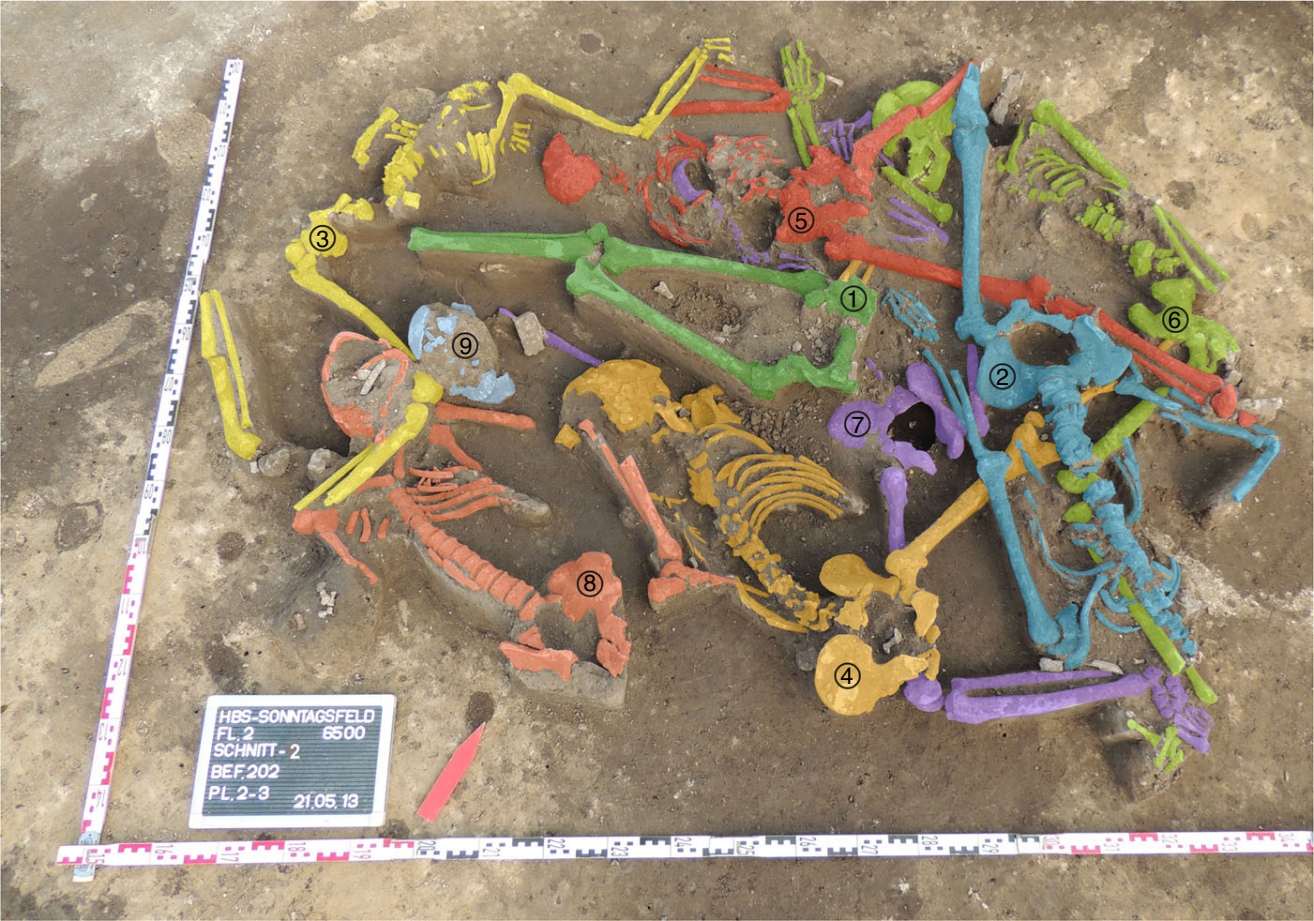
The mass grave feature in situ. Individual skeletons have been coloured and numbered for better visual differentiation

The mass grave was located in the southeastern part of the currently known LBK settlement area at the site. As most other features there, the mass grave pit was partially disturbed by later (pre)historic activities. This made it difficult to precisely define the edges of the actual feature (Fig. 1), which likely had a diameter of about two metres. Apart from the human skeletal remains, the pit contained few other finds, mainly small pottery fragments, some of which show typical LBK ornamentation. These fragmentary finds are interpreted as settlement refuse that accidentally became part of the infill. The individuals in the mass grave were not equipped with durable grave goods. Their body positions differed widely between prone and supine, extended, crouched and irregular with no pattern apparent. All this is in stark contrast to regular LBK burials, including those from the same site^32^, but is very similar to the other LBK mass graves^12^. Six radiocarbon samples of human bone give an overall dating range of 5289–4856 cal bc, which can be refined to 5214–4911 cal bc and probably even to 5080–4997 cal bc, confirming the attribution of the mass grave to the late LBK. This dating range also demonstrates chronological overlap with the nearby settlement, which contains both earlier and later inhumations (Supplementary Table 1; Supplementary Figs 2 and 3).

### Demography

The pit contained nine individuals, seven of them diagnosed as male (78%). The remaining two are a probable male (11%) and a probable female (11%). The youngest male was ca. 16–20 years old, the probable female ca. 21–26 years (Table 1). Although still subadult in biological terms, as epiphyseal fusion was not complete, the youngest male could have been a social adult in Neolithic society^34^. The remaining seven individuals all fall between 25 and 40 years of age. This highly peculiar, almost exclusively adult male demography (89%) deviates significantly from that of the nearby settlement burial site^32, 33^, which shows the standard demography of almost equal numbers of male and female and adult and subadult individuals (Fisher’s Exact Test; sex: *p* = 0.04; age: *p* = 0.03). The absence of children from the Halberstadt mass grave is especially noteworthy, as these usually are numerous in the regular individual and irregular mass burial sites, as well as in other non-standard deposition types^12, 13, 24, 35^. For example, both massacre mass graves of Talheim and Kilianstädten have balanced ratios of subadults to adults from which the Halberstadt mass grave significantly differs (Fisher’s Exact Test; *p* = 0.04 and *p* = 0.05, respectively). The difference to the child-dominated mass grave of Wiederstedt is even more obvious (Fisher’s Exact Test; *p* = 0.00). In contrast, the encountered sex ratio with a pronounced lack of females is reminiscent of the massacre sites of Asparn/Schletz and Kilianstädten^23, 24^. But overall, the mass grave of Halberstadt clearly represents a population sample different from all other attritional or catastrophic LBK burial assemblages known so far. The clear dominance of younger adult males and the absence of children have to be especially stressed, as these characteristics, in combination, indicate important contextual differences from the other sites of LBK mass burial and/or collective lethal violence.

**Table 1:** Osteological characteristics of the individuals found in the mass grave

**Ind**.	**Sex**	**Age (years)**	**Height (cm)**	**ICT**	**IPT**	**ICD**
1	M	25–35	166	(no skull preserved)	—	Possible
2	M	30–40	171	Occipital R	—	Yes
3	F?	21–26	152	(no skull preserved)	—	Yes
4	M	25–35	162	Frontal R, Parietal RL, Occipital RL	—	No
5	M	16–20	—	Parietal RL	—	Yes
6	M	30–40	161	Parietal R	Humerus R	Yes
7	M	25–35	163	Occipital L	Femur R	Possible
8	M?	25–40	—	Parietal L, Occipital L	Humerus L, Ribs L	Possible
9	M	25–40	—	Parietal L, Occipital R	—	No

*ICT* identified cranial trauma, *IPT* identified postcranial trauma, *ICD* identified carnivore damage, *y* years, *M* male, *F* female, *R* right, *L* left

### Palaeopathology

**Fig. 2:**
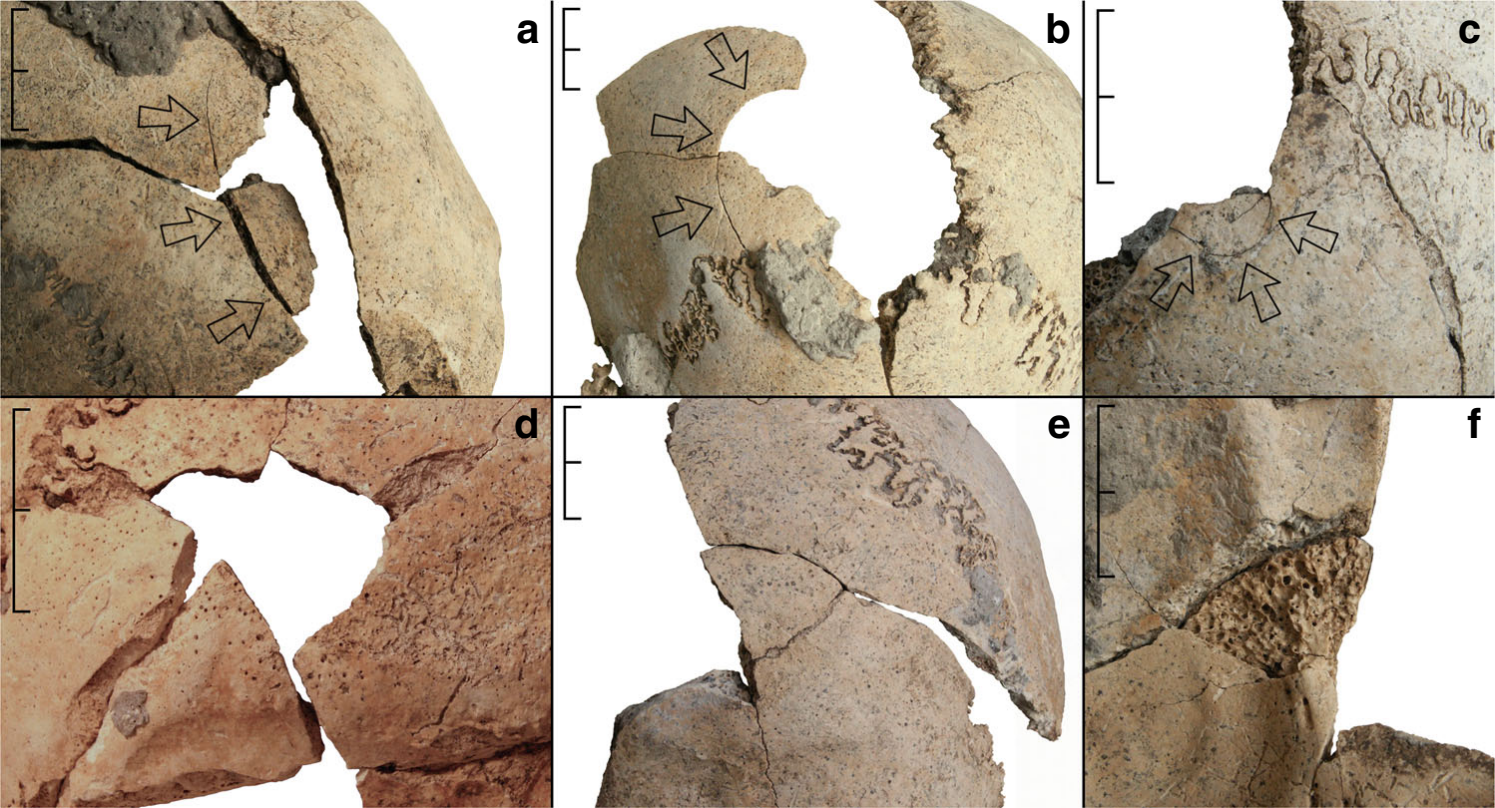
Examples of cranial perimortem blunt force trauma identified in the mass grave. **a** Trauma in the right half of the frontal bone in ind. 4. **b** Trauma in the left parietal bone of ind. 9. **c** Trauma in the midline of the occipital bone (near bregma) in ind. 5. **d** Trauma in the left half of the occipital bone of ind. 7. **e**, **f** Trauma in the right half of the occipital bone in ind. 9. Panels **a**–**e** in ectocranial view; panel f in endocranial view. Scale bar length is 2 cm

**Fig. 3:**
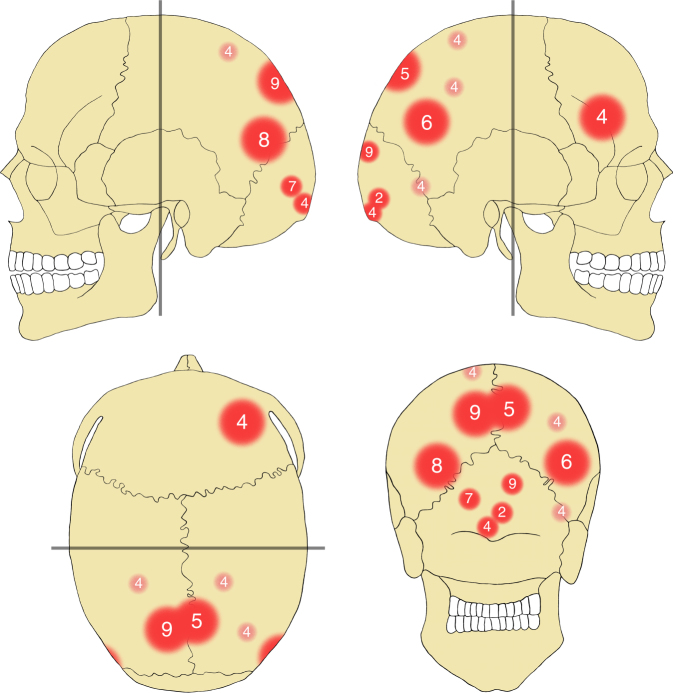
Compilation of the approximate locations of cranial trauma. Signature size represents general trauma size (large vs. small), darker shading represents securely identified trauma, lighter shading represents likely trauma. Signature locations indicate approximate points of initial impact. Numbers indicate the affected individuals with skulls preserved (ind. 2; inds 4–9)

The analysis revealed perimortem blunt force cranial injuries as the most numerous lesions in the assemblage (Fig. 2). All seven individuals with extant cranial remains show at least one perimortem cranial trauma (Table 1). Individuals 5 to 8 have one cranial injury, ind. 9 likely suffered two, and ind. 4 shows at least five or, more likely, six separate traumata. As the skeletal remains are incompletely preserved, it is possible that more injuries had originally been present. Also, due to the fragmentary and fragile condition of some of the bones, cranial trauma has initially been subdivided into securely identified perimortem trauma (9, 69%) and likely perimortem trauma (4, 31%). All instances of the latter are found in ind. 4, who also shows two securely identified perimortem cranial fractures. The recognised injuries as a whole are almost exclusively located in the posterior half of the skull (12, 92%), affecting the posterior parts of the parietal (7, 54%) and the superior parts of the occipital bones (5, 38%) (Fig. 3). Only one perimortem trauma is found on a frontal bone (1, 8%). This injury is again found in ind. 4 and represents one of the securely identified fractures. The locations of the likely injury zones of ind. 4, the most traumatised individual in the sample, are within the same areas as in all other individuals, thereby supporting the overall pattern. Therefore, both trauma categories are pooled again for all further discussion. More injuries are found on the right side (8.5, 65%) than on the left side of the cranium (4.5, 35%), which is also the case at Talheim and Asparn^20–22^. Due to incomplete preservation of fracture margins and missing bone fragments, size and shape could not be precisely determined for all traumatic lesions, but at least two of the best preserved traumata appear very similar in their dimensions (Fig. 2d–f). Both are roughly triangular in shape and are located in the horizontal midline of the occipital bone slightly above the external occipital protuberance and the superior nuchal line. The fracture in ind. 7 is slightly to the left of the median plane and measures ca. 23.4 × 16.8 mm; the fracture in ind. 9 is slightly to the right of the same plane and measures ca. 24.4 × 17.7 mm. Several other fractures with incompletely preserved margins located in the occipital bone appear to have been of roughly similar sizes, measuring between ca. 19.4 and 21.4 mm in one of their dimensions. In general, the identified cranial injuries show the known characteristics of perimortem blunt force trauma, with internal bevelling, concentric and radiating fracture lines, and depressed external fracture margins^36^.

**Fig. 4:**
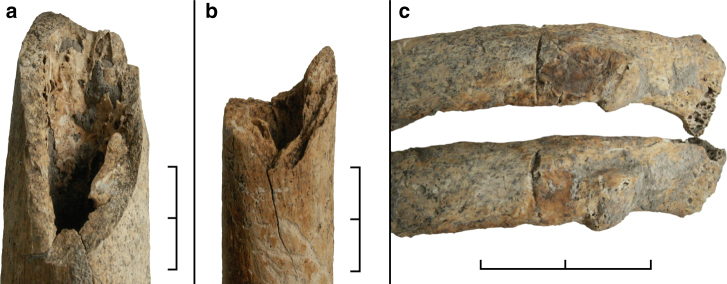
Examples of postcranial perimortem trauma identified in the mass grave. **a** Shaft fracture of the right femur in ind. 7. **b** Shaft fracture of the right humerus in ind. 6. **c** Cutmarks in two left ribs in ind. 8. Scale bar length is 2 cm

In addition, at least three major limb bones of three different individuals show perimortem fractures. The right femur of ind. 7 (Fig. 4a) and the left humerus of ind. 8 are fractured about mid-shaft. The right humerus of ind. 6 has a fracture in the distal third of its shaft (Fig. 4b). All three fractures are complete. Two likely cases of similar fractures are present in one fibula fragment and one forearm bone fragment, both of which had been collected as loose finds from the mass grave prior to the block-lift. One further perimortem trauma has been identified in two neighbouring left ribs in ind. 8. In contrast to all other injuries, these lesions resulted from sharp force trauma and seem to have been caused by a single cut orientated vertically to the axis of the affected bones (Fig. 4c). Besides the perimortem traumata, very few other pathological conditions could be identified, none of which seem to be related to the specific burial context under consideration here. These lesions consist of isolated Schmorl’s nodes in the vertebral column of ind. 2, healed avulsion injuries of lumbar vertebrae 3 and 4 in ind. 8, and a single caries lesion of the lower right first molar in ind. 9.

### Taphonomy

**Fig. 5:**
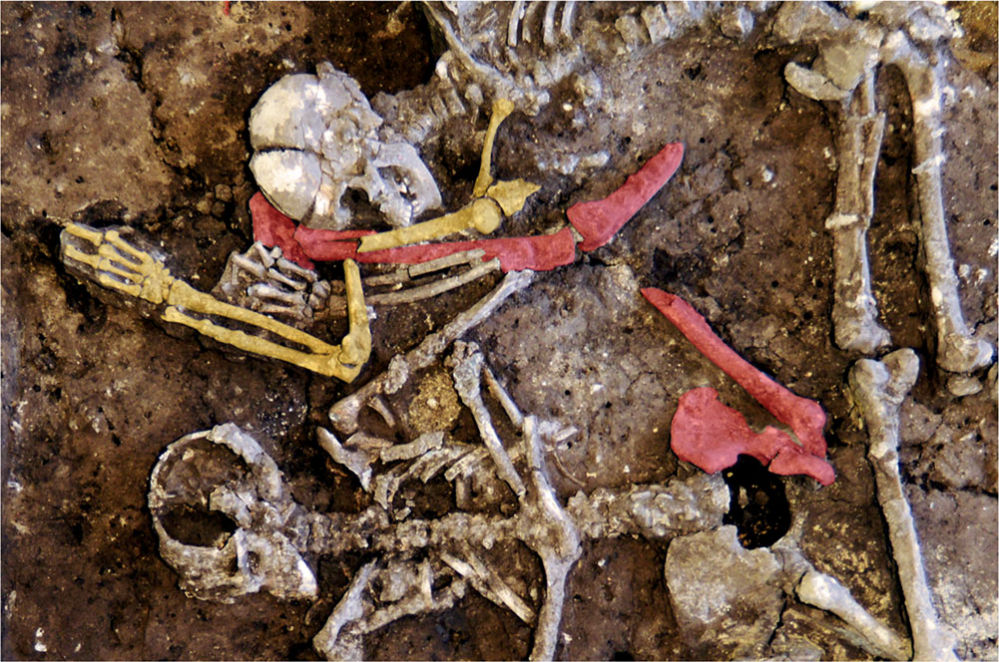
Examples of aberrant positioning of perimortem fractured limbs. In situ positions of the perimortem fractured right upper limb in ind. 6 (humerus; yellow) and right lower limb in ind. 7 (femur; red)

The bodies appear to have been dumped into the pit without care for their individual positions, which differ from those encountered in carefully arranged LBK burials^14, 15^. The spread of the corpses followed the outline of the pit, the limbs apparently remained as they had fallen. This is exemplified most clearly by the aberrant positions of the perimortem fractured arm and leg bones of two of the individuals (Fig. 5). The complete fractures of the shafts acted like additional joints in these cases which resulted in ca. 90° angles between the fracture ends. Their spatial relations also indicate that, although complete osteological discontinuity occurred, soft tissue connections were still in place. In the right femur of ind. 7, the distance between both fracture ends implies that these soft tissue connections were not wholly complete anymore, allowing the proximal end of the distal part of the limb to rotate away from the distal end of the proximal part upon deposition, while still remaining partly attached.

Upon excavation, several major skeletal elements were already missing (Fig. 1). These include the complete skulls of inds. 1 and 3, and most of the skull of ind. 2. The upper half of the body of ind. 1 is absent, as well as its left lower leg bones. Ind. 2 is missing the bones of its right leg and most parts of the left lower leg, while the proximal part of the left tibia is still present and articulated. Ind. 4 is missing the left leg bones, ind. 5 the right lower leg bones, ind. 6 the left leg bones including the left pelvic bone and sacrum, ind. 8 the bones of both forearms, the left femur and all right leg bones. In ind. 9, all bones distal to the fifth cervical vertebra are absent. There is a possibility that the remains labelled ind. 1 and ind. 9 are actually part of the same individual, but the distance between these partially preserved remains and their different orientation rather suggest that they belong to different individuals, as all other major body parts actually present in the feature were still articulated upon deposition.

**Fig. 6:**
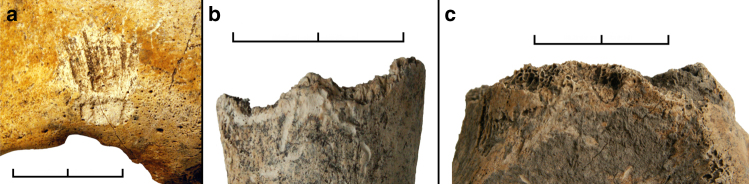
Examples of animal damage to the bones from the mass grave. **a** Typical rodent gnawing damage above the right orbit in the skull of ind. 7. **b** Carnivore gnawing damage to the distal right tibia in ind. 3, partly overlain by root etching. **c** Carnivore gnawing damage to the proximal left tibia in ind. 2. Scale bar length is 2 cm

Many smaller elements, especially of the hands and feet, but also cranial fragments, ribs and vertebrae, are missing as well. These elements have most likely been displaced and removed over time by small burrowing animals. Evidence for rodent activity is manifold, and includes smaller bones and pottery fragments scattered throughout the feature. Infilled rodent tunnels were observed entering the mass grave in different places, rodent skeletal remains were found within the grave fill and several human bones show clear traces of rodent gnawing^37^ (Fig. 6a). Bone damage typical for perimortem carnivore gnawing activity is evident in several individuals^37^ (Fig. 6b, c; Table 1), and is securely identified in skeletal elements along the north-western edge of the mass grave feature. All affected bones are elements of limbs that are missing their distal parts, for example the left tibia of ind. 2 or the right femur of ind. 5. Both of these gnawed bone ends came to rest right at the edge of the feature in the same spot. It is highly likely that the parts missing distal to securely identified carnivore gnawing traces have been removed by these animals. In cases where no carnivore damage was observed, animals might have been responsible for missing bones as well, but later human activity might also have accidentally removed these elements over the millennia. This is quite likely for example for the right leg bones of ind. 8 and the left leg bones of ind. 4. In this area at the southeastern fringe, the edge of the mass grave feature could not be determined anymore, as there is evidence for a later disturbance of significant extent (Fig. 1). As both of these legs have been detached at the hip joint, it can be assumed that the bodies had already been skeletonised when this disturbance occurred. Recent mechanical bone damage is evident in several cases, which affected the topmost layer of bones. This damage can in large part be attributed to the routine preparatory utilisation of heavy machinery during the first steps of the excavation before the presence of the mass grave was known.

### Stable isotope analyses

**Fig. 7:**
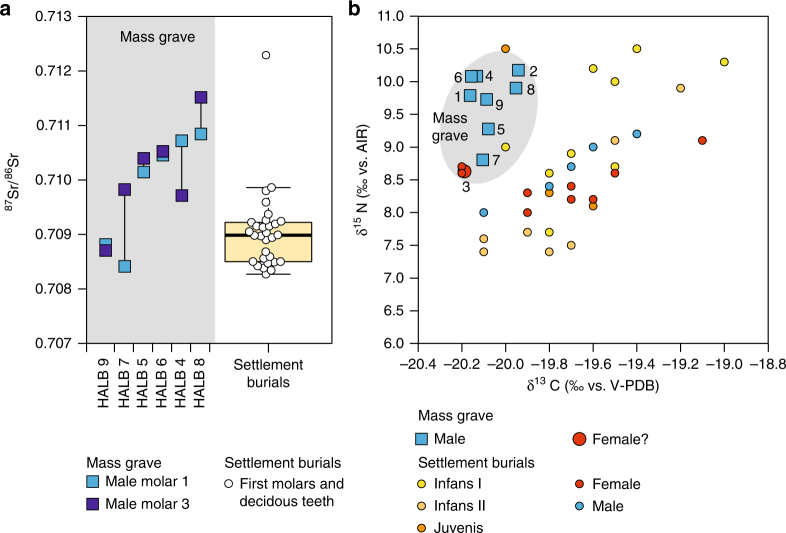
Results of the strontium, carbon and nitrogen isotope analyses. Strontium isotope ratios of enamel (**a**) and carbon and nitrogen isotope data of bone collagen (**b**) of the individuals from the mass grave in comparison to data from regular LBK and likely LBK settlement burials at Halberstadt. Both analytical methods show the mass grave individuals to be different from the regular interments implying both non-local origins and distinct dietary habits. Individuals subsequently identified as non-LBK have been removed from the settlement burial dataset published previously^41^

Strontium isotope analysis (^87^Sr/^86^Sr) on tooth enamel and carbon and nitrogen isotope analyses (δ^13^C and δ^15^N) on bone collagen disclosed non-local origins and distinct dietary habits of the individuals interred in the mass grave in comparison to the regular settlement burials at the same site (Fig. 7; Supplementary Tables 2–4). The isotopic composition of strontium, which substitutes for calcium in the hydroxyapatite, depends on the geologic conditions in the area from where food and drink were obtained during enamel formation in childhood. Isotope ratios that differ from local baseline data reveal non-local individuals, while isotopic differences between subsequently formed teeth point to residential changes during childhood^38^. ^87^Sr/^86^Sr ratios were determined for a first and a third molar each from the six individuals where dentitions were available. First molars represent a time span between birth and about 3 years of age, while the crowns of third molars form between about 7 and 14–16 years^39^.

The ^87^Sr/^86^Sr ratios of the individuals in the mass burial ranged between 0.70841 and 0.71151. While individuals 4, 5, 6, and 8 exhibited more radiogenic ^87^Sr/^86^Sr ratios than most of the settlement burials and individual 7 had a rather large difference of 0.00140 between his first and third molar, only the Sr isotope data of ind. 9 were well comparable with those of the settlement burials as a whole (Fig. 7a). Assuming that the regular settlement burials represent the local population, this suggests that the individuals in the mass grave were largely non-local to the site.

Carbon and nitrogen isotope ratios of bone collagen reflect dietary compositions including the contribution of C_3_ and C_4_ plants, meat and dairy products, freshwater and marine fish^40^, but also manuring^8^. The δ^13^C values of the bone collagen from the individuals in the mass grave ranged between −20.2 and −19.9‰ (vs. V-PDB), while the δ^15^N exhibited values between 8.6 and 10.2‰ (vs. AIR). They form a narrow data cluster of distinctly lower δ^13^C and higher δ^15^N values than the settlement burials^41^ (Fig. 7b). Both the Sr and the C and N isotope data characterise the individuals in the mass grave as clearly distinct from the settlement burial population.

## Discussion

The number of LBK sites showing unusual burial practices and lethal collective violence is slowly but steadily growing^20–24, 28, 35^. The mass grave of Halberstadt represents the most recent addition in this regard that can be placed into the general context of Early Neolithic warfare, defined here as violent organised conflict between independently acting and likely territorial groups^42, 43^. Although the general occurrence of warfare in the Early Neolithic of Central Europe is now largely accepted^25, 44, 45^, its scope and overall impact are still sometimes disputed^18, 19, 46^. It is therefore important to briefly consider the general context in which LBK warfare likely occurred. In the Early Neolithic, political power was not centralised. Rather, local communities, or groups of communities, or maybe even subgroups within settled communities, probably connected by kinship ties regardless of the level of organisation^47^, very likely acted independently from each other as separate political units. Therefore, Early Neolithic warfare as such was necessarily restricted to the same local or regional level and local LBK communities, or possibly alliances of communities, are thought to have been perpetrators and victims alike of the massacres known so far. These also represent the best physical and only direct evidence for actual LBK warfare. In this general context, the massacres have to be taken primarily as separate instances of warfare occurring largely independently from each other and not as part of an all-encompassing, simultaneously occurring pan-LBK conflict. However, as the massacres are not randomly distributed through time, but apparently cluster near the end of the LBK sequence in the decades before 5000 cal bc, it is very likely that they have to be regarded as stark symptoms of profound changes that affected the interlinked social and natural landscapes of large parts of the LBK universe during this time^24, 44^. Climate-induced drops in agricultural production, the mounting consequences of inherited claims to agricultural land and increasing hierarchical differentiation are among the likely factors for furthering the rise of social tensions and, ultimately, of lethal conflicts between independently acting groups^3, 9, 10, 24^.

In addition to the skeletal evidence for perimortem collective lethal violence, as well as possible torture, mutilation, dismemberment and cannibalism^35^, healed injuries are known from skeletons from various LBK burial contexts. These mostly include isolated traces of blunt force and arrow injuries^48, 49^, the same kind of wounds encountered unhealed in higher numbers in the massacre samples. Generally, these healed injuries indicate that violent interactions were not always fatal, but their specific context cannot be reconstructed anymore for individuals interred long afterwards in regular cemeteries^50^. While such wounds may have been received during massacres, either as an active participant or as a surviving victim, they may also stem from interpersonal violence on an individual or familial level. But taken together, there is now ample evidence that collective violence was a major societal issue at least for later LBK populations.

The mass grave of Halberstadt now adds previously undescribed features to LBK collective violence, its context, consequences, and repercussions. The demography is clearly different from the other violence-related mass fatality sites and especially noteworthy is the lack of children. This indicates a context very different from the other sites, as children would undoubtedly have been present in a settlement which came under attack. Children are numerous throughout the known examples of massacres and ritual mass dismemberment^20–24, 35^. This clearly shows that children were not exempt from a violent death but were in fact common casualties^34^. As the Halberstadt mass burial was placed in an LBK settlement area, sound reasons need to be identified for why no children are included. The obvious dominance of younger adult males is equally striking. In contrast to all other sites of LBK regular or irregular burial, the Halberstadt mass grave sample is highly selected towards this group. In fact, the demographic profile rather fits an attacking than an assorted assaulted group^51^. This interpretation is supported by the isotope data, which show that the males in the disorganised mass grave differed from the carefully interred settlement burials regarding geographical origins and dietary habits. Judging from their bioarchaeological profiles, especially the radiogenic ^87^Sr/^86^Sr ratios of their enamel, the killed men can be regarded as non-locals, originating from outside the regular marriage networks and/or recruitment areas of the Halberstadt LBK community.

Massacres and warfare are generally male-dominated activities^30, 45^, and the clear association of weaponry with the male sex supports this view also for the LBK^9^. Furthermore, no significant pathological conditions were found in the skeletal remains that might have had a debilitating effect during life. In contrast, the known Neolithic massacre samples usually contain several individuals handicapped for example by previous traumatic injuries or infectious disease^20, 24, 52^, just like regular attritional burial sites^53, 54^. Such physical handicaps will naturally occur in a cross- or longitudinal section of a settled community as the consequences of an agricultural lifestyle^55^, but not so much in a group of younger men likely chosen for their warfare-related prowess^56^.

**Fig. 8:**
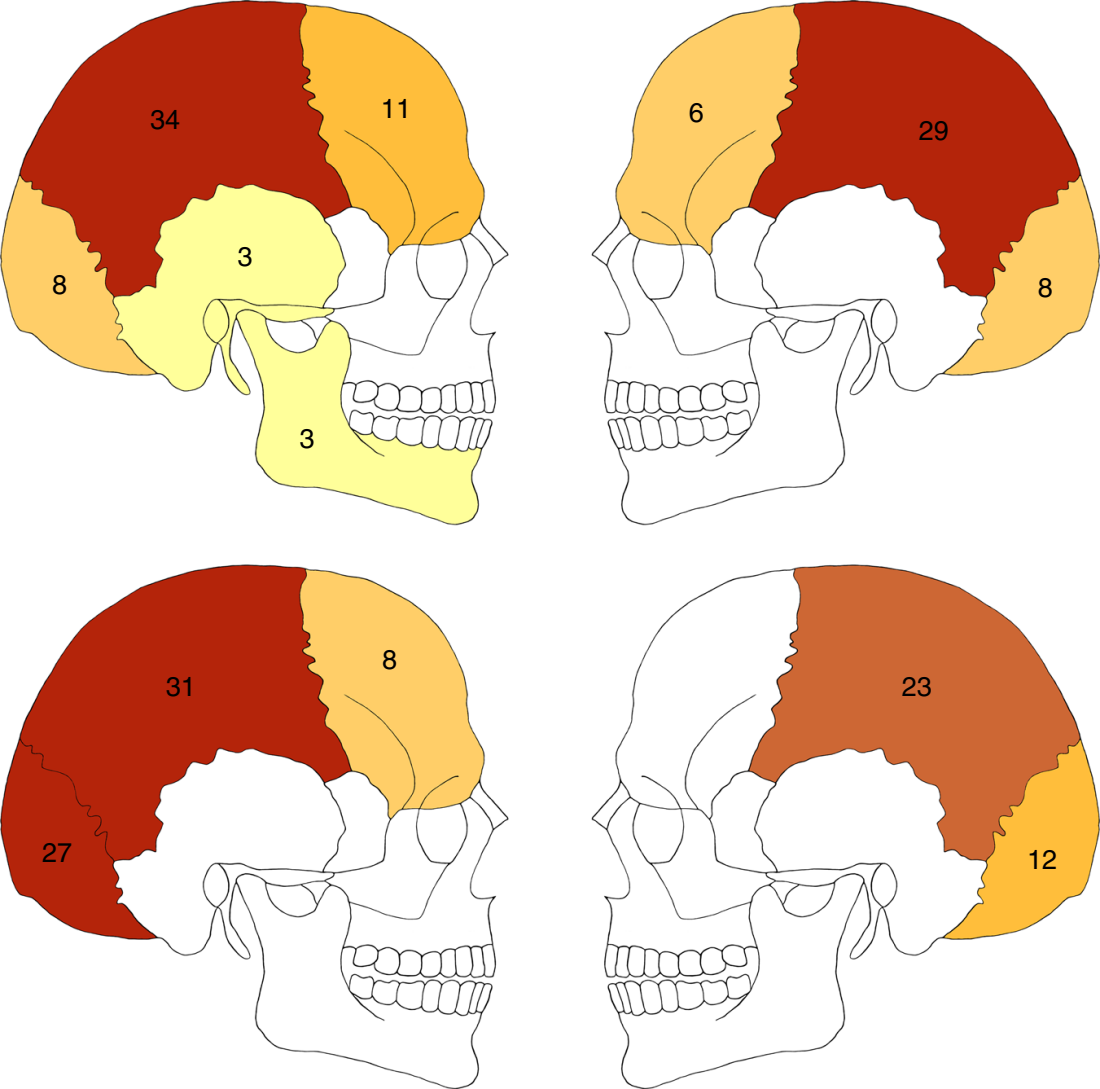
Comparison of perimortem cranial trauma distributions in two LBK mass grave sites. Numbers and respective shading depict approximate percentages of overall blunt force cranial trauma identified at Talheim (top) and Halberstadt (bottom). Talheim data adapted from the literature^70^

Comparing the patterns of perimortem cranial trauma, the Halberstadt sample is again set apart from the previously discovered LBK massacre sites. Injuries cluster almost exclusively at the back of the head at Halberstadt, with only a single deviation from this pattern in the most traumatised individual overall (Fig. 3). Such a tightly circumscribed distribution of wounds is not found at Kilianstädten, Asparn, or Talheim, where more blows hit the frontal than the occipital bones (Fig. 8). Still, it is thought that the victims at Talheim and Asparn were mostly hit from behind, probably while fleeing from their attackers during the chaotic and uncontrolled massacre^20–23^. In contrast, the tightly clustered injury distribution at Halberstadt indicates the deliberate exertion of control and a clearly targeted application of lethal blunt force trauma to the back of the head^57^. The regularity in the placement of the killing blows fits the previously formulated expectations for Neolithic mass execution^21^, which have not been identified at the other mass fatality sites. Although the practice of execution has been suggested previously for the LBK and other Neolithic cultures^35, 58^, firm evidence in the form of standardised cranial injury patterns coupled with a highly selected population sample has so far been lacking.

The killing of people, e.g. captured enemies, under controlled conditions has been a feature of many societies and may have included their previous torture and humiliation^27, 59^. The postcranial perimortem injuries in the Halberstadt individuals may have been caused by such actions, but in contrast to the Kilianstädten sample they are far less common and not as systematic. Alternatively, they may be concomitants of the capture of the individuals with injuries occurring either accidentally or intentionally during a violent struggle^50, 52^.

Carnivore damage has also been found in the skeletal remains at Asparn^23^, which indicates a time of exposure of the bodies. But in contrast to Asparn, the Halberstadt dead were collected into the mass grave a short while after death, as the bodies still retained most of their anatomical integrity upon deposition. As all securely identified cases of carnivore damage in the Halberstadt sample have been found right at the edge of the grave feature in three different places, it is conceivable that the bodies were not covered completely at first. Possibly, the distal parts of some limbs remained accessible to carnivores and were gnawed off at the edge of the feature. Alternatively, the carnivore damage occurred prior to deposition in the mass grave.

The general presence of carnivore gnawing adds to the interpretation that the Halberstadt mass grave sample represents the careless disposal of executed others^31, 60^, individuals that were not part of the local community. They might have been part of a failed and partly captured raiding party attacking the local group that then chose to deny them a regular burial, thereby making a statement out of their violent end^11, 12, 27^. Alternatively, they might have been prisoners brought in from afar, although this does not fit well with the evidence from the other LBK massacre sites, where only women seem to have been captured alive. The lack of postmortem attention given to the bodies rather negates a very significant ritualistic component, as this usually is recognisable in Neolithic contexts either in the careful arrangement or the modification of the remains themselves^35, 61, 62^. Although it may be impossible to differentiate some sort of sacrifice from more pragmatic practises such as execution, the overall evidence gleaned from the mass grave of Halberstadt supports a pragmatic rather than ritualistic interpretation in direct comparison to the other LBK mass fatality sites.

In any case, the controlled and sometimes public killing of individuals or groups carries a serious message about who wields power over life and death^11^. As shown, the Halberstadt mass grave clearly deviates in content and context from the patterns of demography and trauma encountered in other LBK burial or massacre situations and indicates a controlled and systematic killing of a selected group of non-local people^21, 57^. This site therefore adds the previously discussed but so far unrecorded feature of mass execution as a form of culturally sanctioned violence to the known behavioural repertoire of the LBK. Despite being rare and certainly dependent on a suite of predisposing factors, this behaviour may have had a significant impact by solidifying social cohesion and creating an image of a group capable of successful retaliation^27, 63^. While the massacre sites of Talheim, Asparn and Kilianstädten show that warfare and the destruction of whole communities were indeed part of Early Neolithic life, the mass grave of Halberstadt now elucidates further aspects of violent actions and reactions during the time of the first farming culture of Early Neolithic Central Europe.

## Methods

### Excavation and block-lifting

After the mass grave was unexpectedly discovered during regular excavation prior to construction work, the top layer of skeletal remains was exposed on site to assess the overall size and complexity of the burial feature. Already displaced bone fragments were collected by the field team. It was then decided to block-lift the complete feature in one piece to enable a later detailed bioarchaeological dissection of the block-lifted mass grave under controlled indoor conditions. The subsequent further excavation and disassembly of the feature was carried out in the central finds depot of the State Office for Heritage Management and Archaeology in Halle (Saale), Germany and focused on osteologically defined individuals until all skeletal remains and other finds had been removed.

### Osteological preparation and analysis

After the successive removal of the skeletal remains from the block-lifted feature, the adhering loose soil was removed from the bones with cold water and soft toothbrushes. After this step, most bone surfaces were still covered by extensive and irregular layers of compact calcareous encrustations. In part, these were carefully removed mechanically during the drying process at room temperature. In cases where the encrustations could not thus be removed without damaging the bone substance, the bones were soaked in 10% acetic acid until most of the adhering concretions were dissolved, then rinsed again repeatedly with water and dried at room temperature. Overall, encrustations were removed as far as possible from the diagnostically relevant bones to enable a full osteological analysis. Morphognostic methods were used in age and sex estimation of the individuals, as summarised in detail in the standard manuals^39, 64, 65^. Age estimation was based on a summary assessment of skeletal and dental development, maturation, wear and degeneration of all available skeletal elements. Sex estimation primarily made use of dimorphic pelvic features, followed by cranial features and comparative osteometric assessment of the postcranial skeleton. Body height was calculated using the Pearson formulae also assembled for easy use in the standard manuals^65^. Pathological and taphonomical alterations of the bones were assessed in reference to the respective standards of the field^36, 37, 65, 66^. The bone fragments collected prior to the block-lifting process were reattributed to the respective individuals during the osteological analysis as far as possible. Some bones could not be securely reattributed, but these did not raise the number of individuals represented and did not carry significant further information relevant for this study. For statistical testing Fisher’s Exact Test (one-tailed) has been used^67^.

### Strontium isotope analysis

Strontium isotope analysis followed previously established protocols^68^. Enamel samples were cut from tooth crowns and all surfaces and dentine were removed using diamond-coated dental cutting and drilling equipment. The samples were then powdered in an agate mortar, pre-treated with buffered acetic acid and ashed. Sr was separated under clean-room conditions using Eichrom Sr-Spec resin. Sr concentrations were determined by Quadrupole-Inductively Coupled Plasma-Mass Spectrometry (Q-ICP-MS) and ^87^Sr/^86^Sr ratios by High-Resolution Multi Collector-ICP-MS (Neptune) at the Curt-Engelhorn-Centre for Archaeometry in Mannheim, Germany. Raw data were corrected according to the exponential mass fractionation law to ^88^Sr/^86^Sr = 8.375209. Blank values were lower than 10 pg Sr during the whole clean lab procedure. The NBS 987 and Eimer & Amend (E & A) standards run along with the human samples yielded ^87^Sr/^86^Sr ratios of 0.71024 ± 0.00001, 2*σ*; *n* = 5 and 0.70802 ± 0.00001, 2*σ*; *n* = 4, respectively.

### Carbon and nitrogen isotope analysis

Sample preparation for carbon and nitrogen isotope analysis followed previously published protocols^69^ with omission of the ultrafiltration step. Compact bone samples were cut, the surfaces removed, and demineralised in 10 ml of 0.5 N HCl at initially 4 °C and later at room temperature for 14 days, rinsed to neutrality and reacted with 10 ml of 0.1 M NaOH for 24 h at 4 °C, rinsed again to neutrality and gelatinised in 4 ml of acidified H_2_O (pH 2–3) for 48 h at 75 °C. Insoluble particles were separated using EZEE filter separators, and the collagen frozen and lyophilised. C and N contents and the stable isotopic compositions were determined in triplicates using a Thermo Flash 2000 Organic Elemental Analyzer coupled to a Thermo Finnigan Mat 253 mass spectrometer at the Department of Applied and Analytical Palaeontology, Institute of Geosciences at the University Mainz. The raw data were calibrated against the international Standards USGS 40 and USGS 41. Interspersed samples of IAEA CH6 gave a mean δ^13^C value of −10.35 ± 0.01‰ and IAEA N2 gave a mean δ^15^N value of 20.61 ± 0.06‰.

### Radiocarbon dates

Two independent and securely attributed bone samples were collected from inds 2, 3 and 7 each during excavation and were submitted for AMS radiocarbon dating at the Klaus-Tschira-Laboratory for Radiometric Dating Methods, Mannheim, Germany. Results were calibrated and further analysed using the online version of OxCal v4.3/IntCal13 (Supplementary Figs 2 and 3; Supplementary Table 1). Within the text, the 2*σ* date ranges are used.

### Data availability

All relevant data are included in the manuscript and the supplementary information. The human skeletal remains recovered from the mass grave (A6500/D879; feature 100202) are housed at the State Office for Heritage Management and Archaeology of Saxony-Anhalt in Halle (Saale), Germany under catalogue numbers 6500:100202:1-154.

## Electronic supplementary material

Supplementary Information
