# Supplementary material for: Early Neolithic executions indicated by clustered cranial trauma in the mass grave of Halberstadt
Source: Nat Commun. 2018 Jun 25;9:2472. doi: 10.1038/s41467-018-04773-w (PMC6018543; doi:10.1038/s41467-018-04773-w)
Supplement: Supplementary file 1 — Supplementary Information [file 41467_2018_4773_MOESM1_ESM.pdf]

SUPPLEMENTARY INFORMATION

Early Neolithic executions indicated by clustered cranial trauma in the mass grave of Halberstadt

Christian Meyer, Corina Knipper, Nicole Nicklisch, Angelina Münster, Olaf Kürbis, Veit Dresely, Harald Meller, Kurt W. Alt

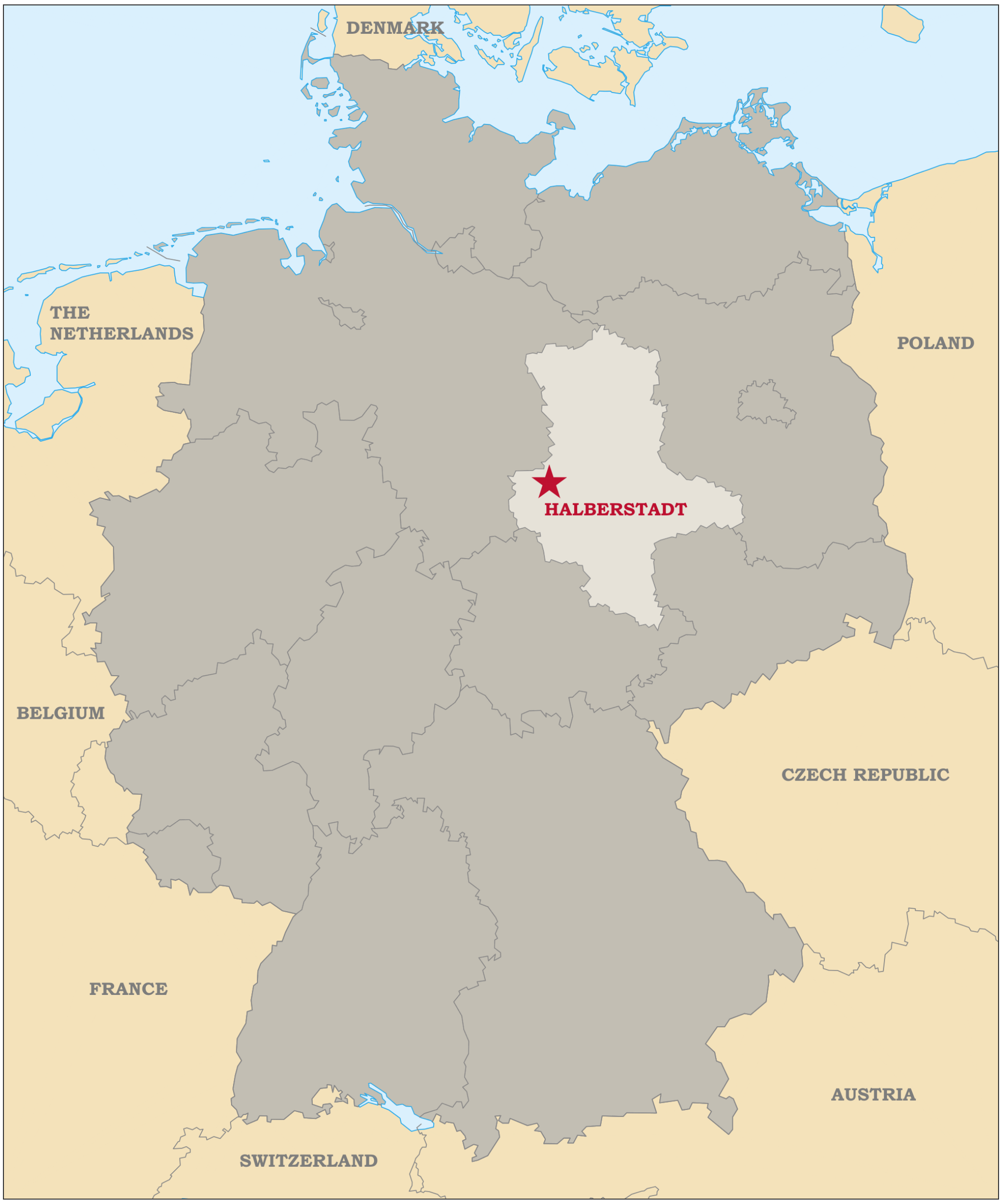

Supplementary Figure 1. Map of Central Europe showing the site of Halberstadt-Sonntagsfeld (star) located within Germany (dark grey) and the German state of Saxony-Anhalt (light grey).

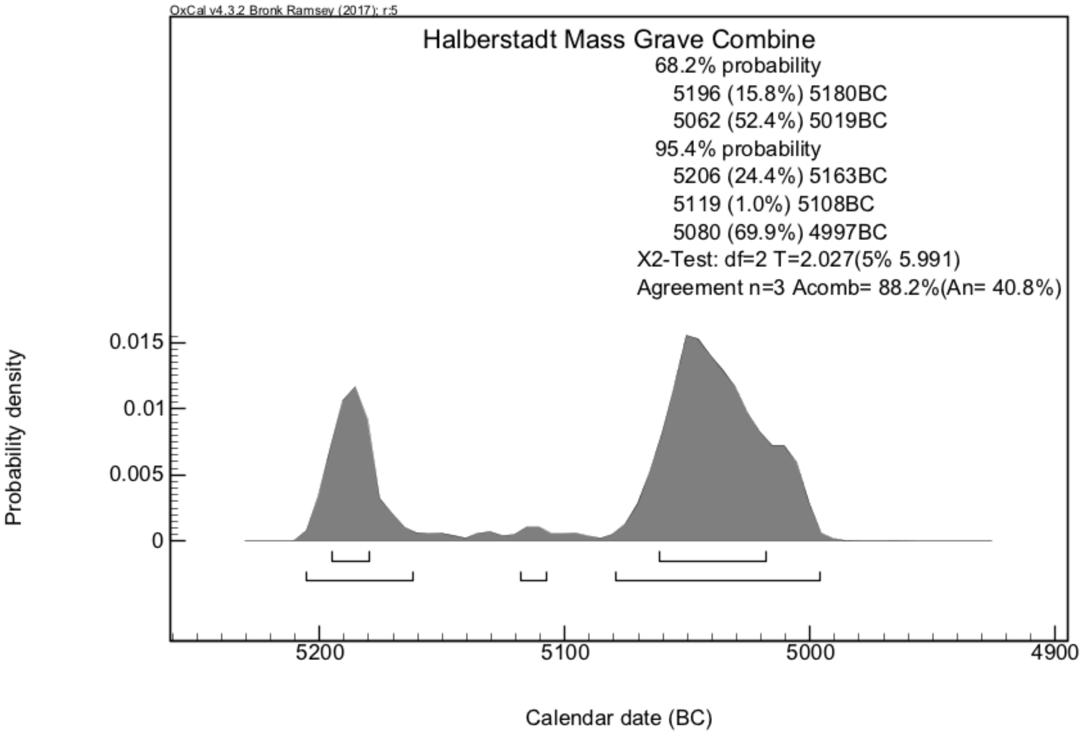

Supplementary Figure 2. Single plot of “combined” radiocarbon dating results of six different bones from three different individuals from the mass grave of Halberstadt (using OxCal v4.3/IntCal13)[1,2].

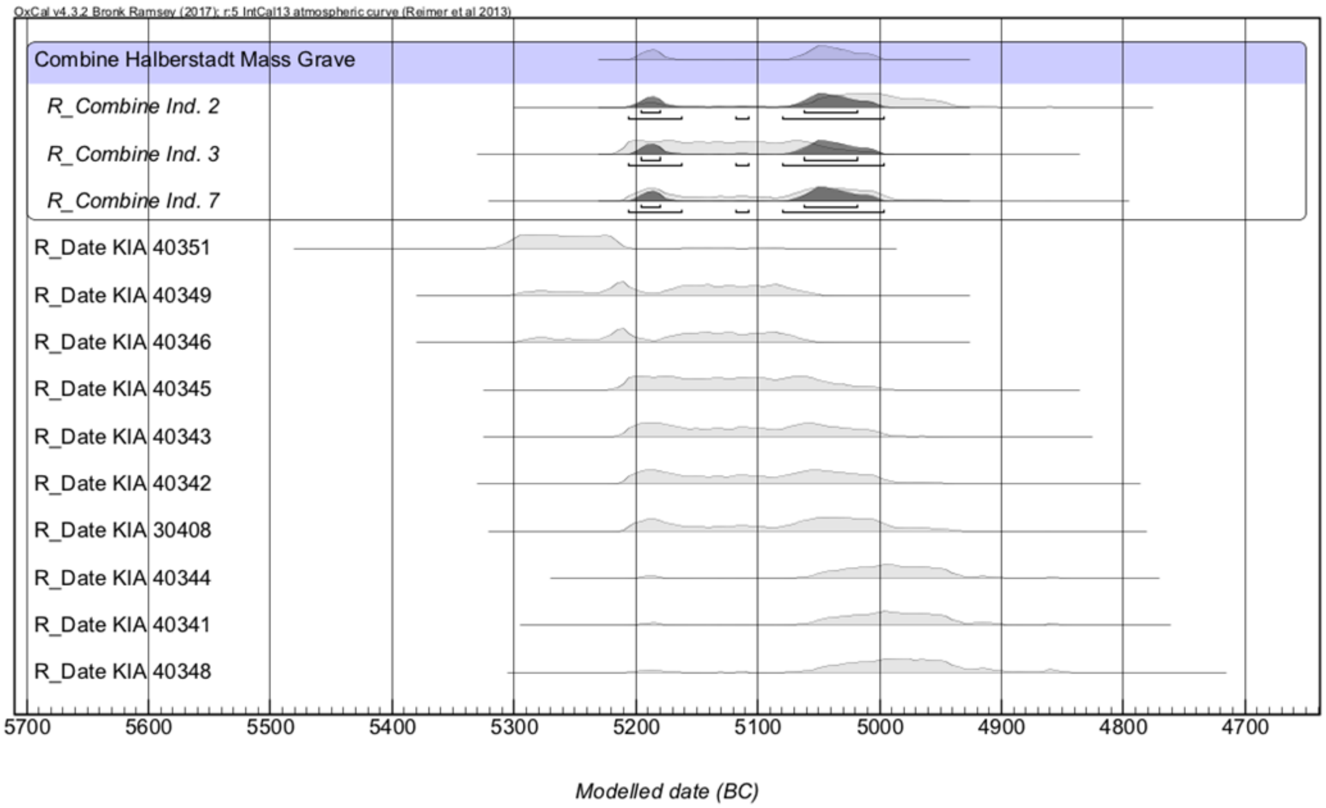

Supplementary Figure 3. Multiplot of "combined" radiocarbon dating results of six different bones from three different individuals from the mass grave of Halberstadt (using OxCal v4.3/IntCal13)[1,2], supplemented by previously published radiocarbon dating results of the regular Halberstadt LBK settlement burials[3].

Supplementary Table 1. Radiocarbon dating results of six different bones from three different individuals from the mass grave of Halberstadt, including OxCal v4.3/IntCal13[1,2] "R_Combine" results for each dated individual. y – years.

Lab no.	Ind.	Sample	14C age, y	±	Cal 1 σ	Cal 2 σ
MAMS 23988	2	Vertebra	6093	42	cal BC 5193-4943	cal BC 5208-4856
MAMS 23989	2	Rib	6096	42	cal BC 5194-4944	cal BC 5208-4859
Combined	2	-	6095	30	cal BC 5051-4962	cal BC 5206-4911
MAMS 23990	3	Rib	6141	42	cal BC 5207-5010	cal BC 5215-4965
MAMS 23991	3	Fibula	6183	42	cal BC 5212-5066	cal BC 5289-5004
Combined	3	-	6162	30	cal BC 5207-5056	cal BC 5214-5024
MAMS 23992	7	Fibula	6134	43	cal BC 5207-5000	cal BC 5213-4962
MAMS 23993	7	Rib	6122	43	cal BC 5207-4988	cal BC 5210-4949
Combined	7	-	6128	31	cal BC 5205-4997	cal BC 5210-4986

Supplementary Table 2. Carbon and nitrogen isotope data of bone collagen of the individuals interred in the mass grave at Halberstadt. The collagen yields, C and N contents and atomic C/N ratios of all samples are in agreement with accepted quality criteria[4].

Ind.	Sample	Collagen yield (%)	C (%)	N (%)	Atomic C/N	$\delta^{13}C$ (‰ vs. V-PDB)	$\delta^{15}N$ (‰ vs. AIR)
1	Pelvis	8.1	43.1	15.3	3.3	-20.16	9.79
2	Rib	5.7	42.8	15.0	3.3	-19.94	10.17
3	Rib	5.1	41.8	14.7	3.3	-20.19	8.63
4	Rib	5.2	42.5	14.9	3.3	-20.13	10.08
5	Rib	7.3	42.9	15.1	3.3	-20.08	9.27
6	Rib	5.6	42.7	15.0	3.3	-20.16	10.08
7	Rib	8.2	43.3	15.3	3.3	-20.10	8.80
8	Rib	5.5	43.1	15.1	3.3	-19.95	9.90
9	Atlas	9.0	42.9	15.1	3.3	-20.09	9.72

Supplementary Table 3. Strontium isotope data of tooth enamel (permanent molars) of the individuals found in the mass grave at Halberstadt.

Ind.	Tooth	$^{87}Sr/^{86}Sr$	2 SD	Tooth	$^{87}Sr/^{86}Sr$	2 SD
1	-	-	-	-	-	-
2	-	-	-	-	-	-
3	-	-	-	-	-	-
4	M 36	0.71071	0.00002	M 28	0.70971	0.00002
5	M 16	0.71014	0.00002	M 18	0.71039	0.00001
6	M 46	0.71045	0.00001	M 48	0.71052	0.00002
7	M 16	0.70841	0.00001	M 18	0.70981	0.00001
8	M 36	0.71084	0.00002	M 38	0.71151	0.00002
9	M 16	0.70881	0.00002	M 18	0.70870	0.00004

Supplementary Table 4. Comparative strontium isotope data of tooth enamel (first molars and deciduous teeth) of settlement burials at Halberstadt.

Lab no.	$^{87}Sr/^{86}Sr$	2 SD	Lab no.	$^{87}Sr/^{86}Sr$	2 SD
HAL_1	0.70894	0.00003	HAL_16	0.70959	0.00001
HAL_2	0.71229	0.00010	HAL_17	0.70980	0.00004
HAL_3	0.70922	0.00003	HAL_18	0.70850	0.00005
HAL_4	0.70915	0.00006	HAL_19	0.70897	0.00006
HAL_5	0.70915	0.00003	HAL_20	0.70903	0.00007
HAL_6	0.70850	0.00003	HAL_21	0.70986	0.00002
HAL_7	0.70919	0.00002	HAL_22	0.70834	0.00004
HAL_8	0.70899	0.00003	HAL_23	0.70838	0.00001
HAL_9	0.70859	0.00002	HAL_24	0.70842	0.00004
HAL_10	0.70898	0.00002	HAL_25	0.70847	0.00004
HAL_11	0.70868	0.00002	HAL_26	0.70905	0.00004
HAL_12	0.70856	0.00005	HAL_27	0.70913	0.00003
HAL_13	0.70890	0.00001	HAL_28	0.70937	0.00006
HAL_14	0.70926	0.00003	HAL_29	0.70827	0.00006
HAL_15	0.70924	0.00002	HAL_30	0.70849	0.00001

Supplementary References

1. Bronk Ramsey, C. Bayesian analysis of radiocarbon dates. *Radiocarbon* **51,** 337-360 (2009).
2. Reimer, P. J. et al. IntCal13 and Marine13 radiocarbon age calibration curves 0-50,000 years cal BP. *Radiocarbon* **55,** 1869-1887 (2013).
3. Brandt, G. et al. Ancient DNA reveals key stages in the formation of Central European mitochondrial genetic diversity. *Science* **342,** 257-261 (2013).
4. van Klinken, G. J. Bone collagen quality indicators for palaeodietary and radiocarbon measurements. *J. Archaeol. Sci.* **26,** 687-695 (1999).
